# On the geoarchaeology of Limyra (SW Anatolia)—new insights into the famous Lycian city and its environs

**DOI:** 10.1002/gea.21781

**Published:** 2020-03-09

**Authors:** Friederike Stock, Martin Seyer, Anika Symanczyk, Levent Uncu, Helmut Brückner

**Affiliations:** ^1^ Department of Biochemistry and Ecotoxicology Federal Institute of Hydrology Koblenz Germany; ^2^ Institute of Geography University of Cologne Cologne Germany; ^3^ Austrian Archaeological Institute Austrian Academy of Sciences Vienna Austria; ^4^ Department of Geography Bilecik Şeyh Edebali University Bilecik Turkey

**Keywords:** ancient city, Eastern Mediterranean, Finike plain, landscape development, paleogeography, sea level

## Abstract

Geoarchives in ancient settlement sites and their environs bear valuable information about Holocene landscape evolution and human–environment interactions. During the last six millennia, sea‐level and coastline changes have had a significant impact on coastal settlements, some of which even had to be relocated. This paper reveals new insights into the spatio‐temporal development of the Lycian city of Limyra. Selected sediment cores were analyzed using a multiproxy approach, combining sedimentology, geochemistry, micropaleontology, and ^14^C dating. When the postglacial sea‐level rise decelerated, a coastal barrier and a deep lake, presumably a lagoon, evolved after the mid‐Holocene. The siltation history of the lake is complex: three coastal peat layers (mid‐4th millennium BC, end of 3rd/beginning of 2nd millennium BC, beginning of 1st millennium BC), indicate periods of semiterrestrial conditions. That they are sandwiched by lake sediments is consistent with new expansion phases of the lake, most likely triggered by coseismic subsidence. There is evidence of a former lakeshore, dated to between 1400 and 1100 BC, with an intentionally deposited layer of anthropogenic remains, now at 5.5 m below the surface. In the mid‐1st millennium BC, the lake silted up, river channels evolved, and people started to settle the area of the later city of Limyra.

## 1 INTRODUCTION

During the Greco‐Roman period, many ancient settlements were situated along the coasts of the Mediterranean Sea. In Turkey, the geoarchaeology of several historical cities has been intensively studied: for example, in Troy (Kraft, Kayan, Brückner, & Rapp, 2003), in Miletos (Brückner et al., 2006; Brückner, Herda, Müllenhoff, Rabbel, & Stümpel, 2014), and in Ephesos (Brückner, 2005; Kraft, Kayan, Brückner, & Rapp, 2000; Stock et al., 2016). Their history is often related to coastline and sea‐level changes.

Sea‐level changes play a major role in the forming of coastal landscapes. Since the end of the last glaciation, global sea level rose for about 120 m. Archaeological remains, such as ancient settlements, harbors, or fish tanks, are sea‐level indicators for the late Holocene (Benjamin et al., 2017; Kızıldağ, Özdaş, & Uluğ, 2012; Marriner & Morhange, 2007; Marriner, Morhange, & Goiran, 2010; Morhange et al., 2013). Along the Aegean coasts, ancient harbors are silted up, harbor installations often submerged for one or more meters (Brückner et al., 2014; Flemming, 1978; Seeliger et al., 2013; Stock, Pint, Horejs, Ladstätter, & Brückner, 2013). The coastline of southern Turkey has also been studied intensively in the last years regarding sea‐level changes: Gulf of Fethiye (Kızıldağ, 2019), Gulf of Hisarönü (Kızıldağ et al., 2012), Kekova Island (Özdaş & Kızıldağ, 2013), southern Turkey (Desruelles et al., 2009), and Antakya Graben (Tarı et al., 2018).

The city of Limyra in southwestern Turkey is situated at the foothill of Toçak Dağı, a part of the southern Taurus Mts., today ca. 4 km inland. Geology and tectonic setting of this mountain range and southwestern Turkey in general, have recently been studied by Elitez and Yaltırak (2016), Görgün, Kalafat, and Kekovalı (2016) and Hall, Aksu, Elitez, Yaltırak, and Çifçi (2014). As for the Finike plain, the sea‐level evolution was investigated by Desruelles et al. (2009), liquefaction in its eastern part by Uyanık, Ekinci, and Uyanık (2013). Ergin, Keskin, Doğan, Kaan Kadıoğlu, and Karakaş (2007) studied the granulometry and the heavy mineral composition of beach sediments. Geoarchaeological research was conducted by Öner (2013) and Öner and Vardar (2018) regarding the evolution of the Finike plain. The sediment cores were, however, neither sedimentologically or geochemically analyzed nor ^14^C‐dated.

Therefore, a geoarchaeological research project of the Austrian Archaeological Institute (ÖAI) and the University of Cologne (Brückner, Stock, & Uncu, 2016a, 2016b; Stock & Brückner, 2017; Stock, Uncu, & Brückner, 2017) aimed at deciphering (a) the environmental changes, especially from the Lycian (ca. 550–330 BC) to the Late Roman (mid‐1st millennium AD) periods; (b) the thickness of the settlement layers; (c) the maximum extension of the former lake; (d) the earthquake chronology; and (e) the spatio‐temporal shifts in the coastline.

## 2 STUDY AREA

The study area is located in southwestern Turkey, topographically ca. 80 km southwest of the city of Antalya, geologically at the junction between the Hellenic Arc and the Cyprus Arc. The geology to the north and northwest of the Finike plain is dominated by the Bey Dağları unit with Mesozoic to Paleogene limestones. The Antalya nappes to the east and northeast are composed of nonmetamorphic bedrock and ophiolites (Şenel, 1997). Quaternary alluvia of the Finike plain have a thickness of at least 26 m (Öner, 2013). Offshore, Eocene sediments fill the Finike Basin (shelf break at ca. 150 m water depth; then down to 3,000 m water depth) (Aksu et al., 2014). Several alluvial fans derive from the surrounding mountains (Öner, 2013).

**Figure 1:**
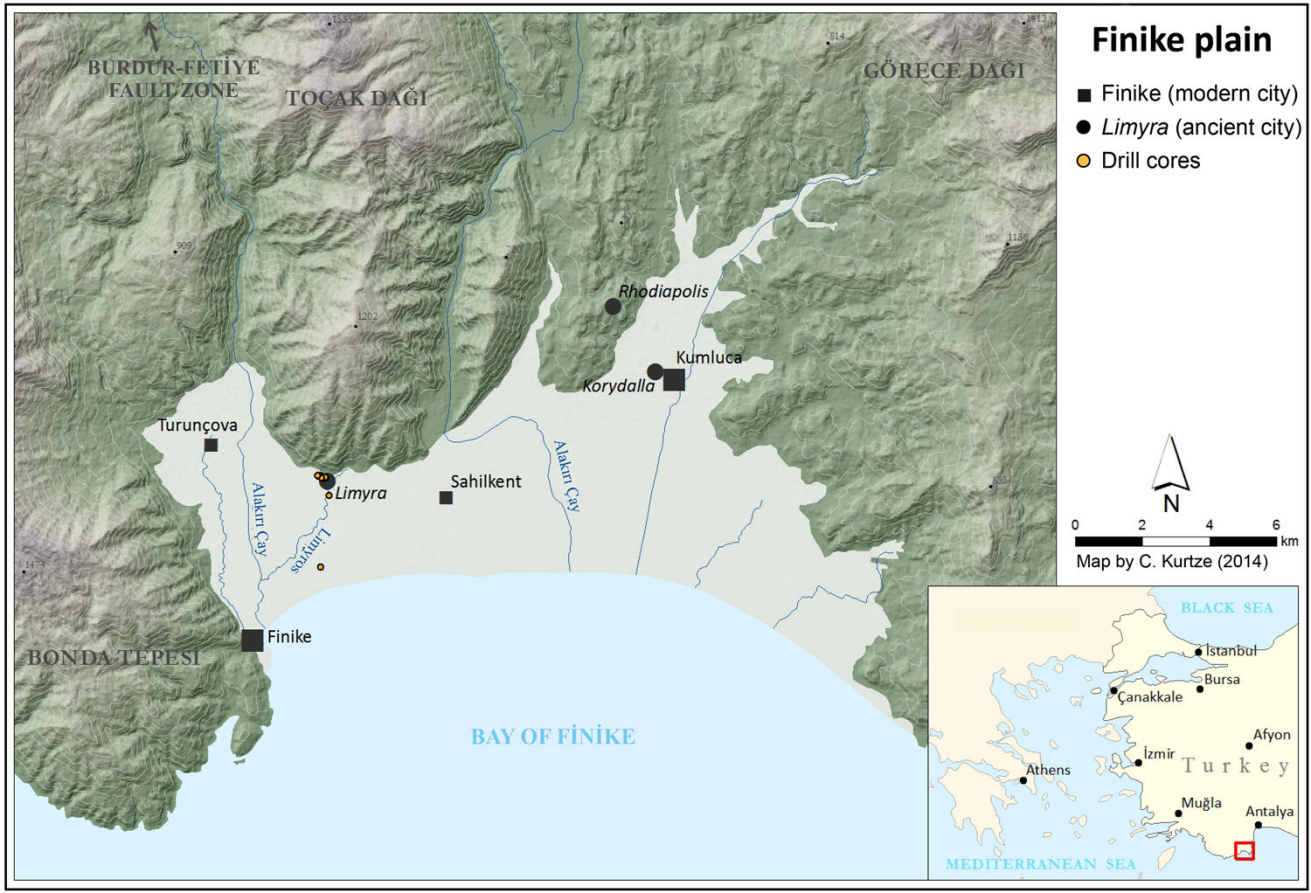
Location of Limyra in the Finike plain (source: Ch. Kurtze, 2014, modified). The locations of some of the drill cores are indicated (yellow dots) [Color figure can be viewed at wileyonlinelibrary.com]

The Finike coastal plain at the foothill of Toçak Dağı (up to ca. 1,200 m; Figure 1) has a W‐E extension of max. 31 km and a width of 7–13 km (Ergin et al., 2007; Figure 1). It evolved despite the enhanced subsidence during the Plio‐Quaternary (including the offshore basin: in total ~5,000 m during the last 5 million years) (Aksu, Hall, & Yaltırak, 2009; Hall, Aksu, Yaltırak, & Winsor, 2009). To the west, the Finike plain is bordered by the Burdur–Fethiye fault zone, which is one of the major faults in Turkey (Hall et al., 2014; Figure 1) generating earthquakes greater than 5.0 (Richter scale; Glover & Robertson, 1998). This explains the frequent occurrence of earthquakes that have already been reported during antiquity (Akyüz & Altunel, 2001; Karabacak et al., 2013). As for Turkey and the surrounding region, Bayrak et al. (2009) attributed the second highest earthquake hazard level to the study area. Based on Öner's (2013) research, who detected an aquatic environment in the subsurface strata, Uyanık et al. (2013) deduced a high risk of earthquake‐generated liquefaction; thus, the 6.8 Mw earthquake of Finike in 1926 would have felt like a magnitude 10 Mw earthquake.

Nowadays, the ruins of Limyra are located ca. 5 km northeast of the modern small town of Finike (Borchhardt, 1993). Several karstic springs rise from the karstified Toçak Dağı immediately adjacent to the north of the city (Bayari et al., 2011). Their waters permanently flow like perennial streams through the ruins, before forming the Limyros (Akçay) river (Öner, 2013).

## 3 ARCHAEOLOGY

The city was first mentioned in Hittite texts (ca. 1350–1300 BC) as *zumarri*. *Zẽmuri*, the Lycian name of Limyra, can with some probability be traced back to this term (Keen, 1998). The earliest archaeological finds date to the early 7th century BC. A settlement existed in this area at the latest since the 6th century BC (Konecny & Marksteiner, 2007). The city experienced its first heyday in the 4th century BC when it became the residence city of an aspiring east Lycian dynasty under the reign of Pericles.

By then, an extensive building program took place in Limyra, during which a massive ring of city walls, including a fortification on the acropolis, was erected. The residential quarters were enlarged, including the lower slope of the acropolis hill with houses partially hewn out of the bedrock. At this time the monumental “heroon” of the dynasty was erected on the acropolis (Borchhardt, 1976). This magnificent royal tomb is a counterpart to the famous, slightly older Nereid Monument from Xanthos. It was designed like a Greek temple with four larger than life‐sized caryatids at each facade. The tomb was raised up above a high podium in which the tomb chamber was situated. The five necropoleis of the Classical period, located in the direct vicinity or close‐by the city, host ca. 400 tombs; this is by far the largest number of tomb buildings of all Lycian cities (Borchhardt & Pekridou‐Gorecki, 2012; Kuban, 2012).

**Figure 2:**
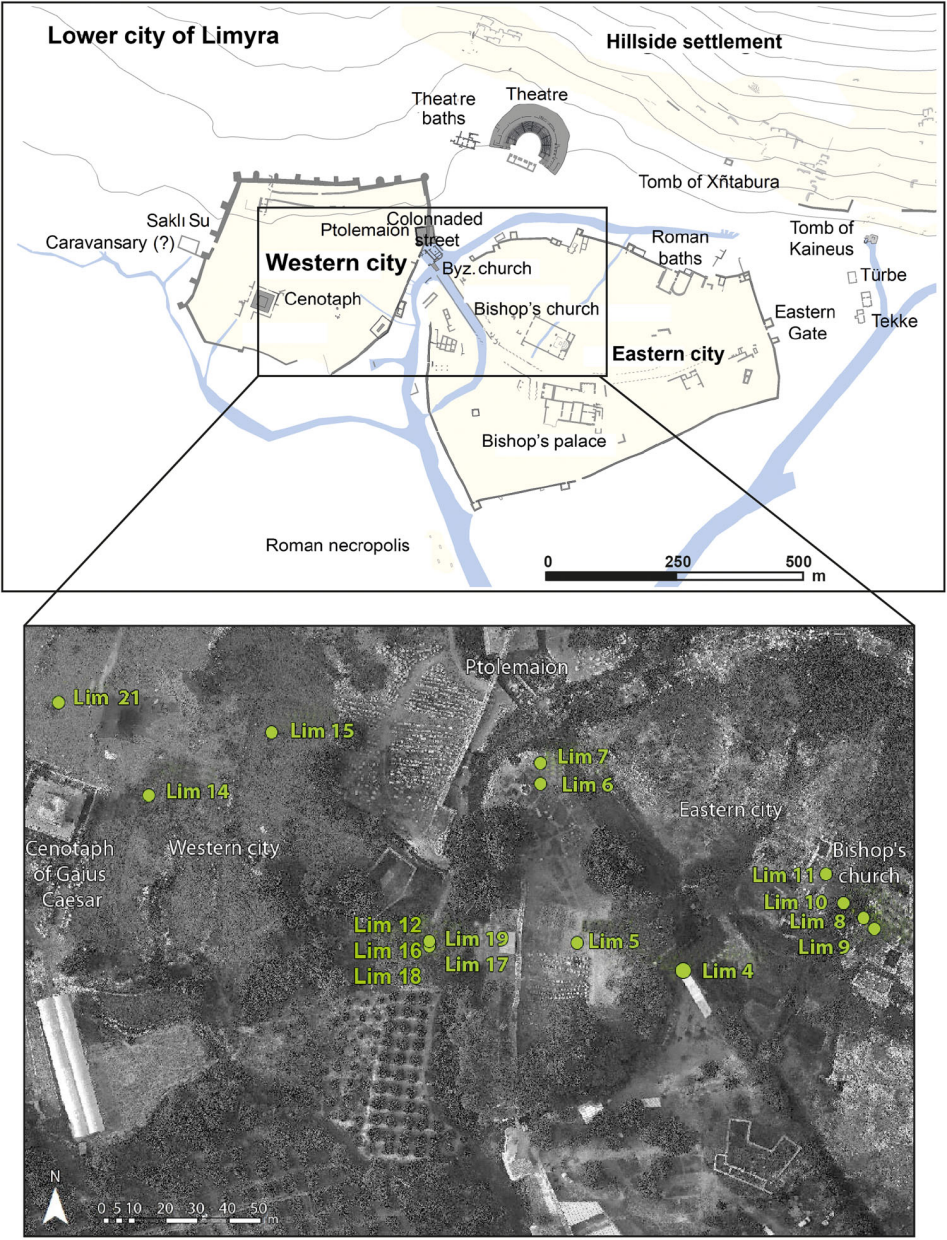
Upper part: Lower city of Limyra with the western and eastern city parts with important buildings (plan: C. Kurtze, © ÖAI Grabung Limyra, modified). Lower part: Location of the drill cores in the city of Limyra (aerial photograph: C. Kurtze, ÖAI, modified) [Color figure can be viewed at wileyonlinelibrary.com]

The Hellenistic and Early Roman periods are only sporadically attested in Limyra by few structures; nevertheless, due to their monumentality and the high quality of their architecture and sculptural decoration, they attest to the importance of the settlement during these epochs. The so‐called Ptolemaion, located in the lower city, belongs to this group of impressive structures (Figure 2). It was set up by the Egyptian dynasty of the Ptolemies in the first half of the 3rd century BC; due to its excellent craftsmanship, it has to be regarded as one of the most outstanding monuments from this period in Asia Minor (Stanzl, 2012; Stanzl, 2016). The most prominent building from the Early Roman Imperial period is the cenotaph of Gaius Caesar, grandson and adopted son of the Roman Emperor Augustus, who died in Limyra on 21 Febuary, AD 4 (Borchhardt, 2002; Ganzert, 1984). Its massive core of *opus caementitium* is preserved in the western lower city of Limyra. The pedestal was originally faced with a 60 m long frieze of marble plaques with life‐sized figures, displaying scenes from the life of Gaius Caesar. Besides the theater, built in the 2nd − 1st century BC and partly rebuilt after a devastating earthquake in AD 141, other buildings—such as huge bathing complexes, broad colonnaded streets, bridges, and so forth—also illustrate the flourishing urban life of Limyra in the Roman Imperial period (Seyer, 2016).

During Late Antiquity and the Byzantine period, the city was an episcopal see. Between the 4th and the late 9th centuries AD, the names of six bishops are known. In the late 5th/early 6th century AD, the city was divided into two parts by means of two separate wall circuits. Amongst the monuments of that time, the three church buildings are noteworthy (Jacobek, 1993; Peschlow, 1984; Pülz, 1996), as well as a building discovered in 2012 that might have been the synagogue of the city (Seyer, 2014; Seyer & Lotz, 2014).

## 4 METHODS

### 4.1 Fieldwork

Our research is based on 21 sediment cores (Lim 1–Lim 21) with a max. length of 12 m. They have been retrieved from Limyra's city center and its environs with a vibracorer Cobra pro (Atlas Copco) in semiopen augerheads (outer diameters: 6, 5, 3.6 cm; Figure 2). Representative samples were taken from different layers. Four master cores were chosen from the study area and analyzed in detail. All sites were leveled with a DGPS (Topcon) (precision: ±2 cm in all three dimensions). The geoarchaeological research design is according to Brückner and Gerlach (2020).

### 4.2 Laboratory analyses

In the laboratory, the sediment samples (59 in total with a resolution of up to 25 cm intervals per drill core) from the four master cores were dried, carefully pestled, and sieved (2 mm). For grain size analysis, they were treated with hydrogen peroxide (15% H_2_O_2_) to destroy the organic matter and with sodium pyrophosphate (Na_4_P_2_O_7_, 46 g/l) for dispersion. The samples were measured with a laser diffraction particle size analyzer from Beckman Coulter (LS‐13320, 116 classes from 0.04 to 2,000 μm) and statistically analyzed with the Gradistat software (Blott & Pye, 2001).

A Bartington MS2 sensor was used for measuring the magnetic susceptibility (MS). To determine the C/N ratio, the samples were homogenized with a ball grinder (Retsch MM 400), weighed in tin boats and measured with a vario EL cube (Meyers & Teranes, 2001). After the determination of total carbon (TC) and nitrogen (N), the total inorganic carbon (TOC) was measured after treating each sample with 10% HCl. X‐ray fluorescence measurements were carried out with a portable XRF device (Niton™ XL3t GOLDD+).

Selected samples from different layers were chosen for microfaunal species determination (ca. 5 g sediment; sieves with mesh sizes of 63 and 100 μm). Ostracods were determined according to Meisch (2000). However, only one sample each from Lim 4 and Lim 14 contained ostracods.

### 4.3 Chronology

Fourteen samples (charcoal, plant fragment, wood, seed) from Lim 4, 10, 12, 14, and 16 were radiocarbon‐dated (AMS‐^14^C) (Table 1). All ages were calibrated with Calib 7.1 (Reimer et al., 2013) and processed with OxCal (Bronk Ramsey & Lee, 2013). They cover a time span from 3700 BC to AD 200.

**Table 1:** Radiocarbon dating results

Sample ID	Lab number	Material	Depth (m b.s.)	Depth (m b./a.s.l.)	^14^C age BP	Age cal BC/AD
Lim 12/16 Pf	UBA‐33133	plant, seed	2.95	1.48	1917 ± 31	AD 4–208
Lim 12/21 Pf	UBA‐33134	plant	5.34	−0.91	2819 ± 31	1056–896 BC
Lim 12/22 H	UBA‐33135	wood/plant fragment	5.46	−1.03	2270 ± 34	400–209 BC
Lim 12/25 T	UBA‐34116	peat	6.7	−2.27	3606 ± 43	2123–1785 BC
Lim 12/29 T	UBA‐34115	peat	10.24	−5.81	4827 ± 33	3693–3525 BC
Lim 16/4 Pf	UBA‐34122	plant	5.5	−1.07	2938 ± 31	1230–1028 BC
Lim 16/5 H	UBA‐34117	wood, charcoal	5.6	−1.17	2857 ± 30	1115–929 BC
Lim 10/14 H	UBA‐34119	plant, wood	3.41	1.62	3000 ± 39	1389–1115 BC
Lim 10/20 T	UBA‐34118	plant	5.31	−0.28	2547 ± 31	801–549 BC
Lim 14/8 HK	UBA‐34120	charcoal	5.83	0.88	3417 ± 35	1874–1626 BC
Lim 4/14 Hk2	UBA‐34123	charcoal	2.85	2.41	2300 ± 39	412–208 BC
Lim 4/19 Hk	UBA‐34121	charcoal	3.72	1.54	2350 ± 35	536–368 BC
Lim 4/29	UBA‐34124	plant	6.43	−1.17	2823 ± 40	1113–857 BC
Lim 4/34	UBA‐34125	plant	8.33	−3.07	3481 ± 37	1896–1692 BC

*Note*: Dating was carried out by the ^14^Chrono Centre for Climate, the Environment and Chronology at the Queen's University Belfast, UK (lab code: UBA). All ages were calibrated with Calib 7.1 (Reimer et al., 2013), and processed with OxCal (Bronk Ramsey & Lee, 2013). They are presented with 2 sigma confidence interval. According to the DGPS measurements by C. Kurtze all altitudes which refer to sea level are corrected for +0.93 m as compared to the reference system of the Limyra excavation. Abbreviations: b.s., below surface; b./a.s.l., below/above present mean sea level.

### 4.4 Statistical analyses

Principal component analysis (PCA) was done with the Past software (version 3.11) for differentiating the facies (Hammer, Harper, & Ryan, 2001). All values of the four master cores were standardized and standard deviation calculated. Ten components—Fe, K, clay, sand, C/N, sorting, mean, CaCO_3_, Ca/Sr, magnetic susceptibility—were used for the PCA.

## 5 RESULTS

Four master cores (Lim 4, 12, 13, and 14) were chosen from Limyra and its environs to decipher the different facies of the Finike plain.

**Figure 3:**
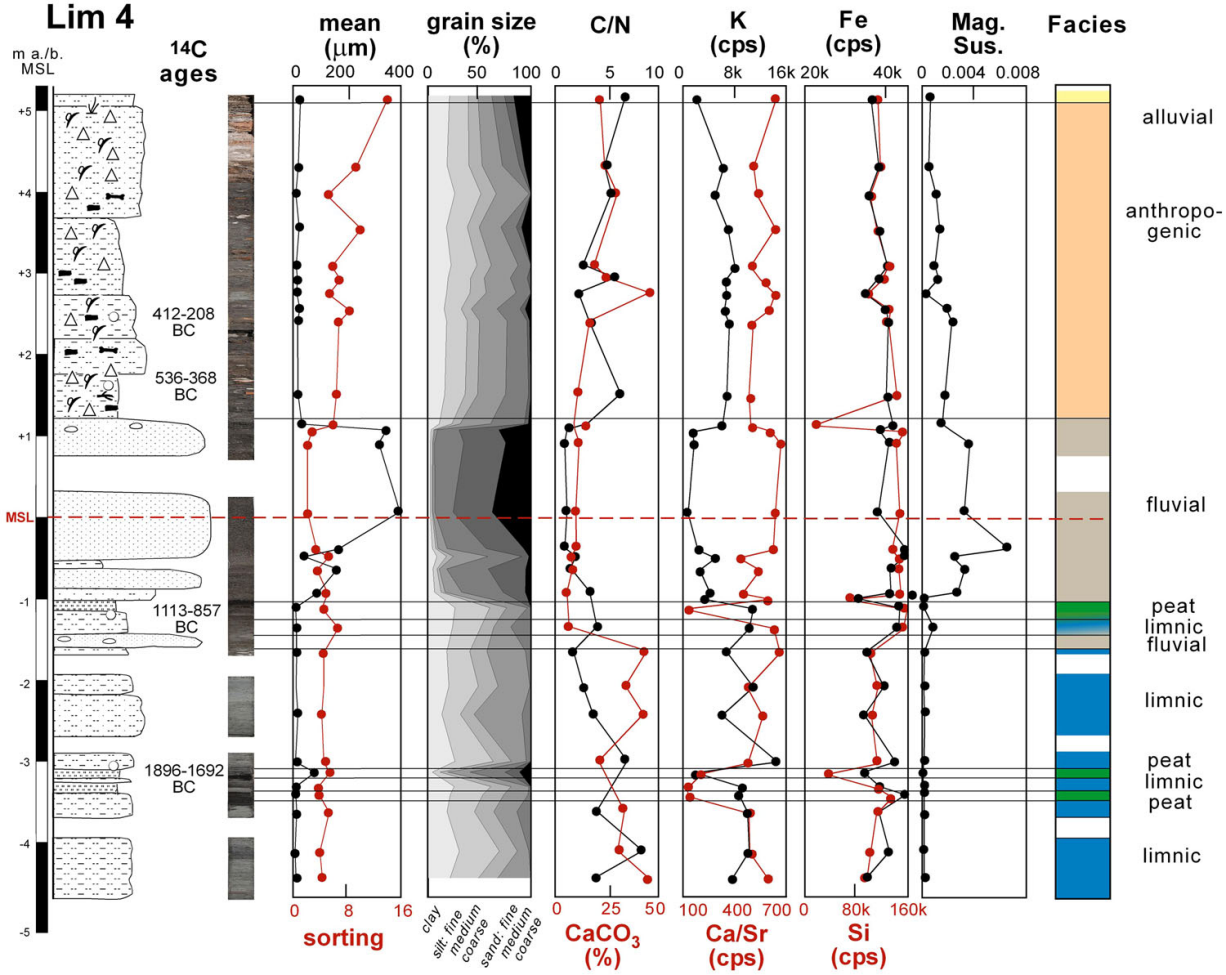
Stratigraphy of Lim 4 with sedimentology, geochemistry, radiocarbon ages, and facies interpretation [Color figure can be viewed at wileyonlinelibrary.com]

### 5.1 The city area

Lim 4 was cored in the northwestern part of the eastern city (surface 5.25 m above sea‐level (a.s.l.); max. depth 10 m below surface (b.s.); Figures 2, 3 and 5). The base is characterized by homogeneous light grayish silty clay with a mean of 3–13 μm, C/N values of 2.6–8.4 and juvenile specimens of the freshwater ostracod *Candona* sp. (4.75–1.45 m b.s.l.; below sea level). Three peat layers are interdigitated (3.53–3.38, 3.23–3.20, 1.10–1.05 m b.s.l.; Figure 3). The fine‐grained sediments on top of the second peat layer date to the first half of the 2nd millennium BC (Lim 4/34, ^14^C age 3481 ± 37 BP, 1896–1692 cal BC), the third peat layer developed during the first half of the 1st millennium BC (Lim 4/29, ^14^C age 2823 ± 40 BP, 1113–857 cal BC). The overlying sediments consist of gravel at the base, covered by sands and alluvia (mean: 37–340 μm), while the uppermost four meters are composed of sandy silt (mean: 6–21 μm) with abundant ceramic, brick and bone fragments, charcoal, as well as core‐filling layers of angular stones (mostly limestone). The C/N ratios differ strongly (0.9–3.3 and 2.2–6.8, respectively). According to the ^14^C chronology, the central part of the uppermost layers dates from the 6th to the 3rd centuries BC (Lim 4/19 Hk, ^14^C age 2350 ± 35 BP, 536–368 cal BC; Lim 4/14 Hk2, ^14^C age 2300 ± 39 BP, 412–208 cal BC).

**Figure 4:**
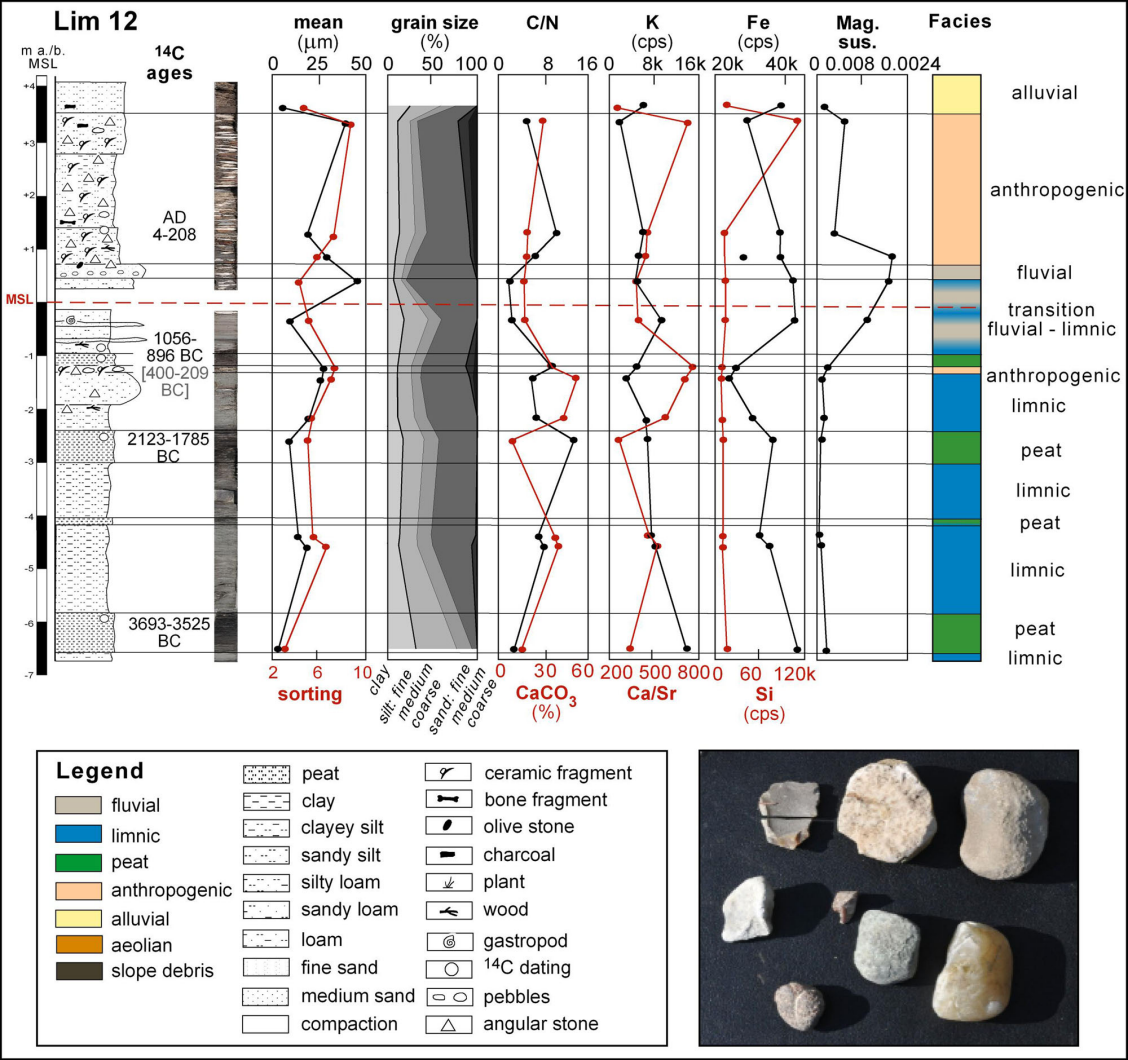
Stratigraphy of Lim 12 with sedimentology, geochemistry, radiocarbon ages, and facies interpretation. Photo of silex (flake?), a processed stone and erratic, nonlocal pebbles found in the anthropogenic layer of Lim 12 at a depth of 1.17–1.07 m b.s.l. (photo H. Brückner). Legend also for Figures 3, 5, 6, 7, 8, 9 [Color figure can be viewed at wileyonlinelibrary.com]

**Figure 5:**
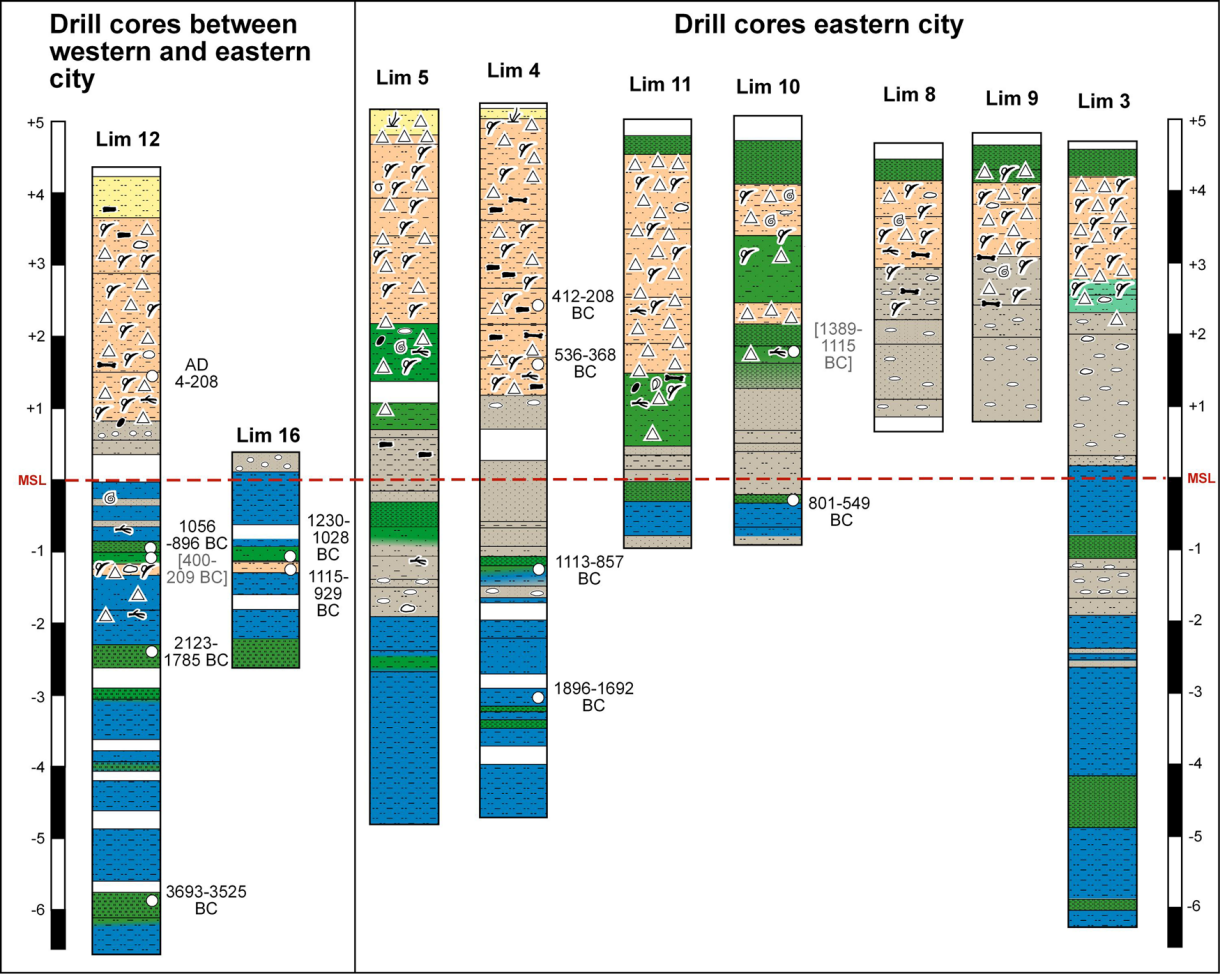
Synopsis of the drill cores from the eastern city (Lim 5, 4, 11, 10, 8, 9, and 3), and drill cores located between the eastern city and the western city (Lim 12 and 16). As for Lim 16, only the section 2.57 m b.s.l.–0.43 m a.s.l. was recovered to learn more about the layer with anthropogenic impact at a depth of ca. 1.15–1.00 m b.s.l. (cf. Figure 4) [Color figure can be viewed at wileyonlinelibrary.com]

Lim 12 is situated between the western and the eastern city (surface: 4.43 m a.s.l., max. depth: 11 m; Figures 4 and 5). This coring confirms the stratigraphy of Lim 4 in its lower part with fine‐grained sediments (clayey silt, mean: 13–18 μm) and three intercalated peat layers. Two age estimates date the lowermost peat to the early mid‐4th millennium BC (Lim 12/29 T, ^14^C age 4827 ± 33 BP, 3693–3525 cal BC), and the one above to the end of the 3rd/beginning of the 2nd millennium BC (Lim 12/25 T, ^14^C age 3606 ± 43 BP, 2123–1785 cal BC). The following grayish silty sands (1.57–1.07 m b.s.l.) are coarsening upwards. After a sharp contact, a layer with angular stones and pebbles of different origin (limestone, chert, quartz) occurs. Moreover, silex (flake?) and a processed stone were found (Figure 4). Then follows peat that is overlain by silts with many stones and ceramic fragments. At a depth of 1.48 m a.s.l., a plant seed was dated to the beginning of the 1st millennium AD (Lim 12/16 Pf, ^14^C age 1917 ± 31 BP, cal AD 4–208).

### 5.2 Drill core Lim 14 in the western city

**Figure 6:**
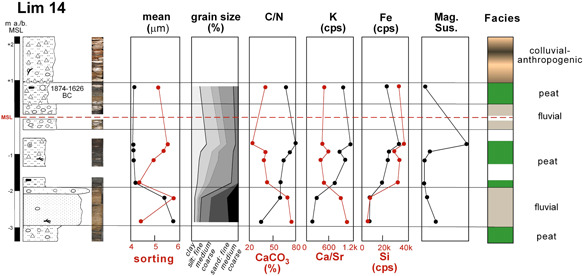
Stratigraphy of Lim 14 with sedimentology, geochemistry, radiocarbon ages, and facies interpretation. The uppermost stratum continues to the surface (6.71 m a.s.l.; not shown here). It is composed of anthropogenic material, mixed with colluvium. Therefore, no sedimentological or geochemical analyses were carried out [Color figure can be viewed at wileyonlinelibrary.com]

**Figure 7:**
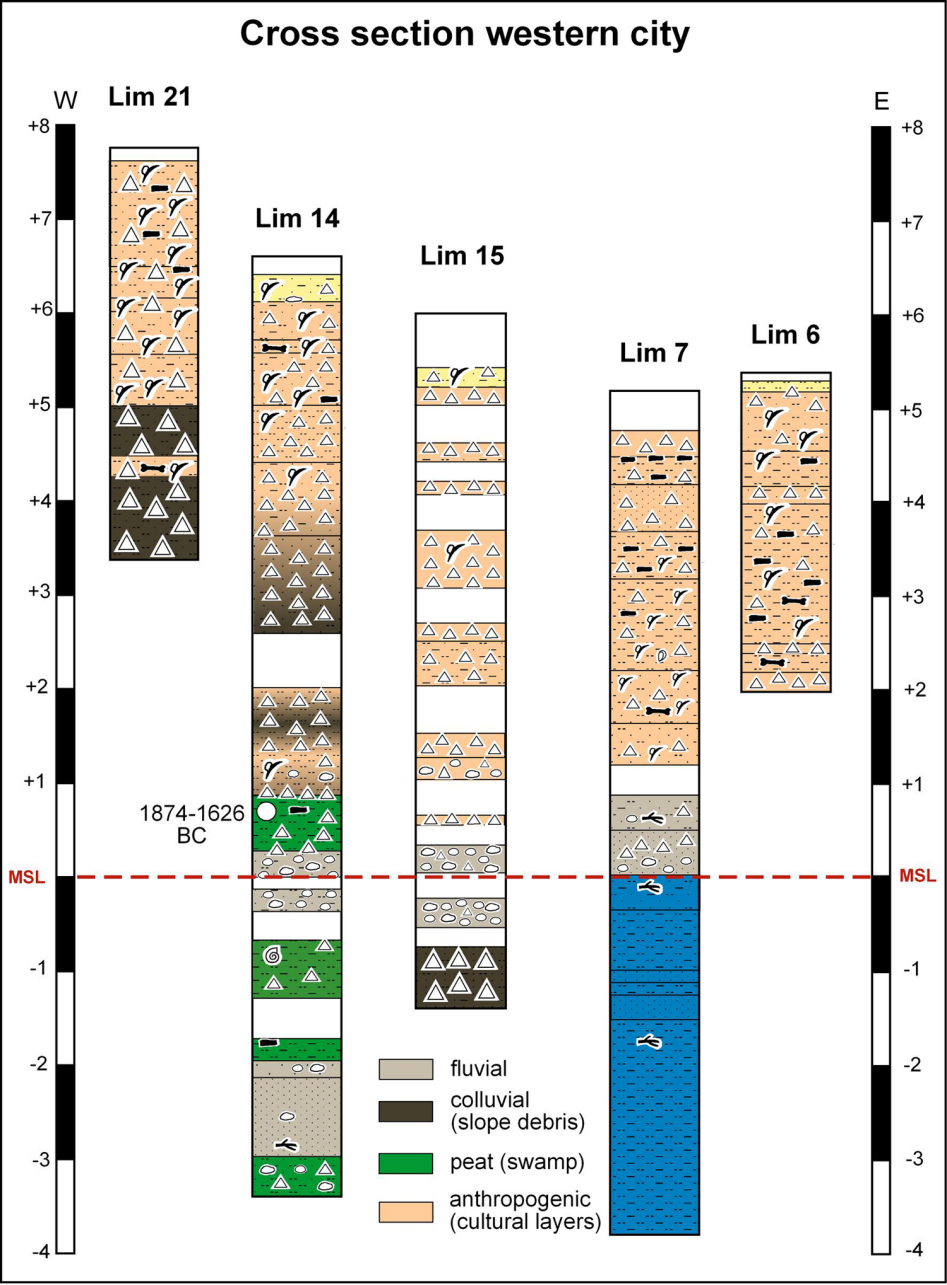
Synopsis of the drill cores within the western city (Lim 21, 14, 15, 7 and 6) [Color figure can be viewed at wileyonlinelibrary.com]

Lim 14 is located in the western city close to the cenotaph of Gaius Caesar (surface: 6.71 m a.s.l., max. depth: 10 m; Figures 2, 6 and 7). At its base, alternating organic‐rich clayey silts (mean 5–18 μm, C/N 5‐8) and calcium carbonate‐rich yellowish‐brown sands (mean 160–210 μm, C/N 2‐5) occur. The transition to the overlying strata dates to 1874–1626 cal BC (Lim 14/8 HK, ^14^C age 3417 ± 35 BP). In this layer, few ostracods (juvenile *Candona* sp., *Ilyocypris bradyi*, *Eucypris* sp., *Darwinula stevensoni*) were found. The uppermost 7 m are characterized by angular limestones (up to 5 cm), fragments of ceramics and bones as well as pieces of charcoal. No analyses were conducted in these anthropogenic layers, since no natural sediment occurred between the core‐filling stones.

### 5.3 The natural sedimentation outside the city area

**Figure 8:**
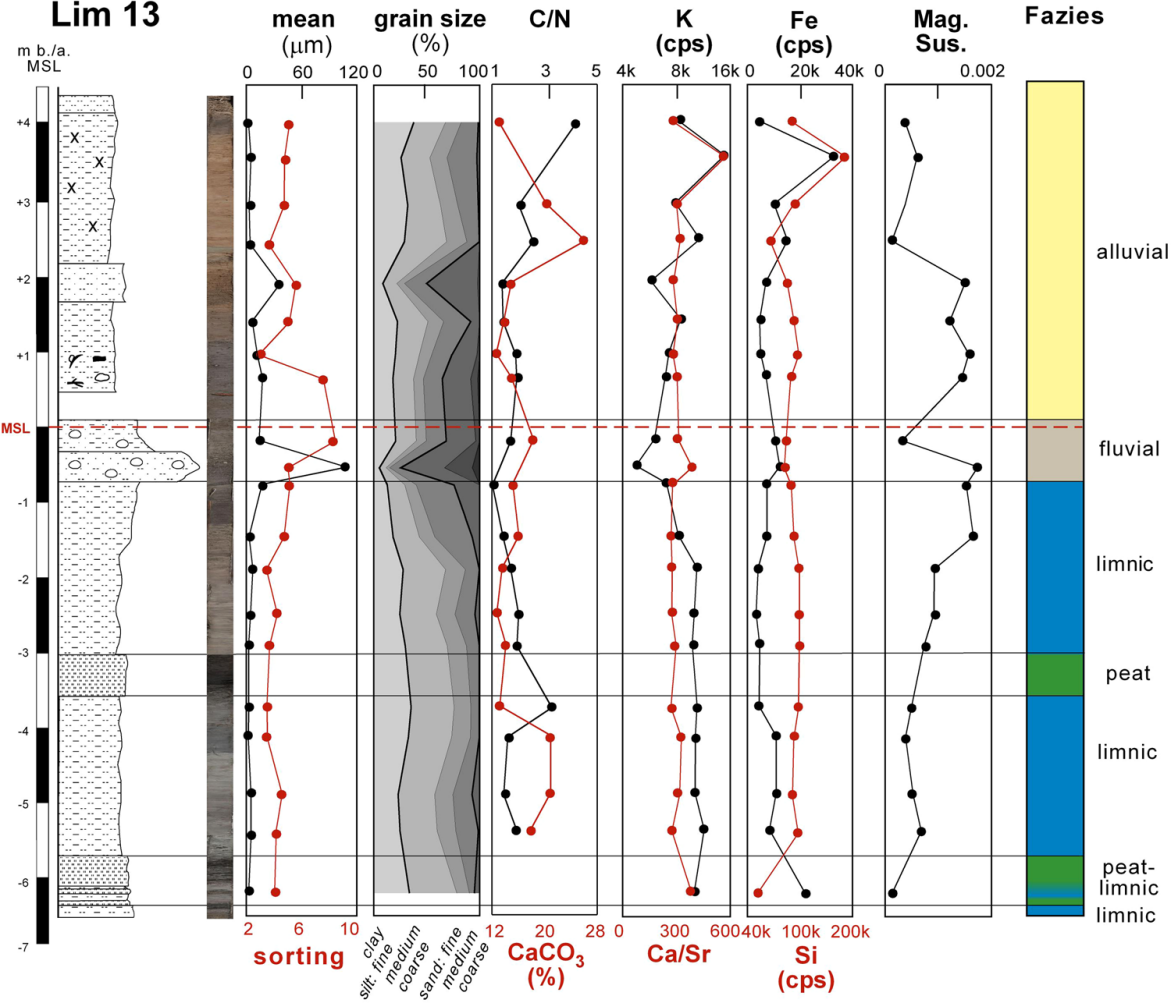
Stratigraphy of Lim 13 with sedimentology, geochemistry, and facies interpretation [Color figure can be viewed at wileyonlinelibrary.com]

**Figure 9:**
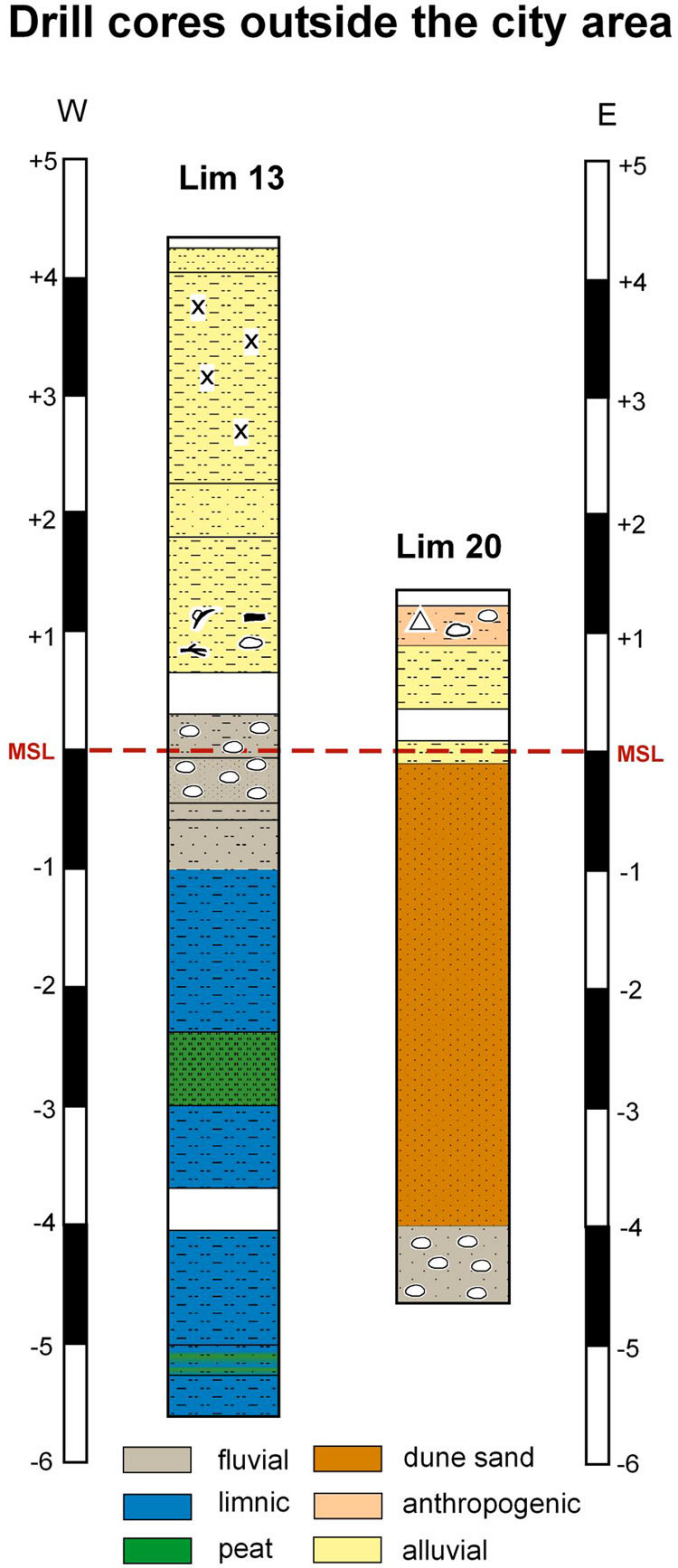
Drill cores Lim 13 and 20 outside the city area. Lim 13 is located ca. 800 m southeast of Limyra, Lim 20 ca. 1 km inland from the present coastline [Color figure can be viewed at wileyonlinelibrary.com]

Lim 13 (surface 4.32 m a.s.l., max. depth: 10 m b.s.) is located 500 m to the southeast of the city area and 50 m to the east of the Limyros river (Figures 1, 8 and 9). The base is composed of homogeneous grayish clayey silts (mean: 3–8 μm, C/N 1–2) with two intercalated peat layers. A sharp contact separates this unit from a sandy layer with pebbles (<1 cm, mean: 118 μm). It is overlain by homogeneous brown clayey silts (mean: 3–7 μm; with the exception of one coarser layer) reaching up to the present surface. A ceramic fragment was found at a depth of 0.83 m a.s.l.

Lim 20 (surface: 1.34 m a.s.l., max. depth: 6 m b.s.), cored near the present coastline, consists of medium to coarse sand with pebbles at the base (4.66–4.31 m b.s.l.), followed by four meters of homogeneous medium sand. As in Lim 13, the uppermost part consists of fine‐grained brown silts.

## 6 FACIES INTERPRETATION

**Figure 10:**
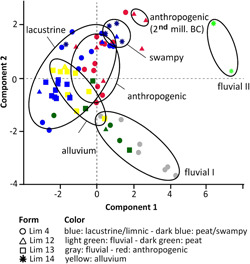
Principal component analysis (PCA) of sediment cores Lim 4, 12, 13, and 14 with ten PCs (mean, sorting, clay, sand, C/N, CaCO_3_, Fe, K, Ca/Sr, magnetic susceptibility). It is based on components 1 and 2 with a variance of 39.7% and 25.5%, respectively (PC 3: 11.5%, PC 4: 7.7%, PC 5: 6.4%, PC 6: 3.8%, PC 7: 2.8%, PC 8: 1.7%, PC 9: 0.9%, PC 10: 0.5%) [Color figure can be viewed at wileyonlinelibrary.com]

The samples of the four master cores represent nine different facies in the Limyra area. They were differentiated by granulometry (mean, sorting), C/N, K, Fe, CaCO_3_ values, and magnetic susceptibility (Figure 10, PCA). The strata of other cores could also be attributed to these facies.

### 6.1 Slope debris facies

This facies occurs in the three cores from the western city close to the mountain before bedrock was reached. It is characterized by many angular stones (mostly limestones), some of which are even bigger than the augerhead (i.e., originally bigger than 5 cm). They derive from the adjacent mountains. In the upper parts of the cores, the slope debris is often mixed with anthropogenic material (pieces of ceramics, bricks, mortar, bones).

### 6.2 Swampy/amphibic facies

The brown organic‐rich silts in Lim 14 are characterized by a high C/N ratio (6–8) and CaCO_3_ content (20–40%) as well as a sorting of 5–6 (swampy; Figure 10). The PCA clearly reveals a distinction to all other sediments. Sorting and C/N point to a low‐energy deposition in an aquatic environment (Kaushal & Binford, 1999). The high Fe (20–30 k cps) and K (8–12 k cps) values together with freshwater microfossils *Candona* sp., *Ilyocypris bradyi*, *Eucypris* sp., *Darwinula stevensoni* also refer to an aquatic origin, probably characterized by shallow water with many plants (Meyers & Teranes, 2001). Moreover, the Si content reflects allochthonous detrital input (Davies, Lamb, & Roberts, 2015).

### 6.3 Lacustrine facies

The homogeneous gray clayey silts containing the freshwater ostracod *Candona* sp. were deposited in a lacustrine environment; they are present in almost all drill cores. The PCA shows a low‐energy sedimentation in a sheltered area most probably reflecting a former lake in the Finike plain. Moreover, the relatively high Fe (ca. 20 k cps) and K (ca. 10 k cps) contents point to input from the surrounding areas (Davies et al., 2015). Towards the top of the layer, the rising values of magnetic susceptibility and granulometry indicate the transition to the fluvial layer (Stock et al., 2016; Figure 8).

### 6.4 Peat layers

In total, four peat layers occur, which are sandwiched by lacustrine strata. As expected, they are all characterized by high C/N ratios of 8–14. The preservation of organic material points to anoxic conditions (Turney, Canti, Branch, & Clark, 2005). Moreover, shallow water is necessary for the development of peat (Kuzucuoğlu, Dörfler, Kunesch, & Goupille, 2011). Since only a few selected samples were measured, they do not cluster well.

### 6.5 Anthropogenic layers

The upper meters of the drill cores from the city area consist of relatively badly sorted silts (mean up to 43 µm) with ceramic fragments, bones, charcoal, and angular stones. The heterogeneity of the anthropogenic layer is reflected in the PCA: most of the samples do not cluster well (red color in Figure 10). Due to their small size and weathering effects the ceramic fragments could not be dated. However, several ^14^C age estimates exist (a) for Lim 4 spanning from the second half of the 6th to the end of the 3rd centuries BC (Figure 3); and (b) for Lim 12 spanning from the beginning of the 1st to the beginning of the 3rd centuries AD (Figure 4).

There is a special layer with strong anthropogenic input, much deeper, however, than the just described ones. It was discovered in several drillings between the two parts of the city at a depth of about 5.50 m b.s., that is ca. 1.10 m b.s.l. (e.g., Lim 12 and 16, Figure 5) with an average thickness of 10–15 cm. A synopsis of the ^14^C ages from this layer and the topping peat leads to the conclusion that it was deposited in the second half of the 2nd millennium BC, roughly between 1400 and 1100 BC. This presumably Late Bronze Age layer is at least 500 years older than the first so far detected archaeological finds.

### 6.6 Fluvial facies

Medium to coarse sands with pebbles were encountered in all drill cores with a thickness varying from 0.50 to 2.50 m (fluvial I; Figure 10). The PCA of this facies is dominated by magnetic susceptibility due to detrital input from the surroundings, a high mean (50–400 µm) and coarse granulometry (ca. 80% of sand).

Two samples from cores in the western city are different from the other fluvial samples (fluvial II; Figure 10), in that their medium to coarse sands are dominated by high values of Ca/Sr (1 k cps) and CaCO_3_ (80%). They occur at greater depths than the other fluvial strata, were deposited before the mid‐2nd millennium BC, and probably represent an old river channel.

### 6.7 Alluvial facies

In the cores outside the city area (Lim 13 and 20; Figure 9), fine‐grained homogeneous silts dominate the uppermost parts of the sediment column. They are void of artefacts and were deposited during the last millennium. Obviously they originate from the flooding of the area in connection with heavy rainfall events after the settlement had been abandoned.

## 7 DISCUSSION

### 7.1 Paleoenvironmental changes during the Holocene

#### 7.1.1 From the mid‐Holocene to the beginning of the 1st millennium BC

**Figure 11:**
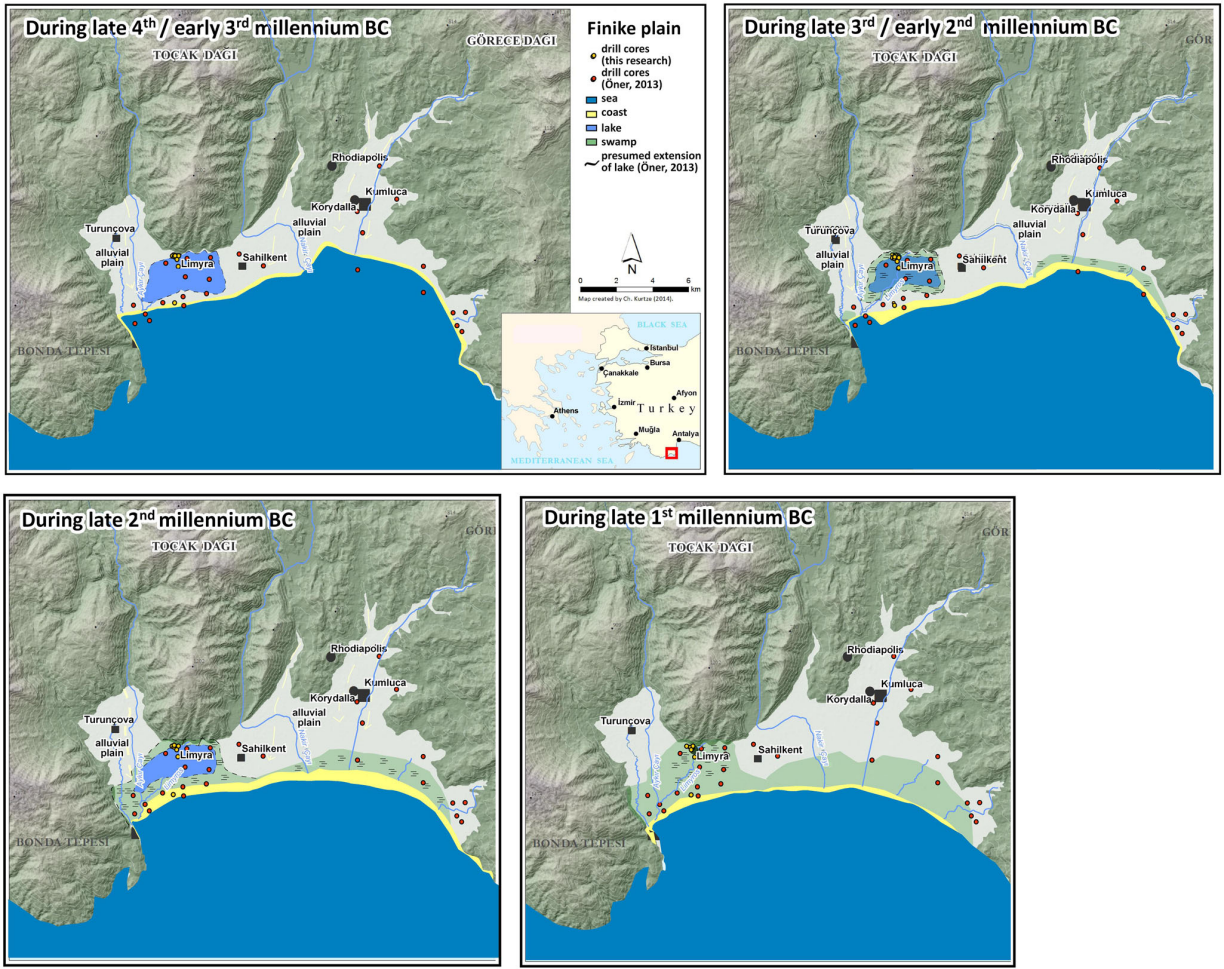
Landscape evolution of the Finike plain shown in four time slices. The scenarios are based on Öner (2013) and our own results [Color figure can be viewed at wileyonlinelibrary.com]

The results presented in this paper are a contribution to deciphering the landscape evolution in the environs of Limyra. In their lowermost section, the drill cores in the western city are composed of slope debris from the adjacent mountains. Then, a lake formed in the (later) Finike plain, starting during the mid‐Holocene at the latest. Lacustrine sediments with a maximum thickness of 7 m were detected in the cores between the western and the eastern city and to the east (Figure 11). The oldest ^14^C age estimate dates to the mid‐4th millennium BC (Lim 12, Figure 4). From our research in other coastal areas of Anatolia (e.g., Brückner et al., 2006; Brückner et al., 2014), we presume that the Holocene stratigraphy starts with deposits from the late Pleistocene—early Holocene marine transgression, that is, shallow marine facies, which then turned into lagoonal facies as evidence of a beach barrier–lagoon complex. However, in the Finike plain marine strata have not yet been encountered. Due to the steep slope gradient of the adjacent mountains (Toçak Dağı), they are obviously at a much deeper depth than the range we can cover with our equipment. Moreover, the strong longshore drift of the sea will have closed the lagoon, which then turned into a freshwater lake due to the strong perennial discharge of the karstic springs. This is why we only encountered limnic freshwater facies. It is thus most probable that the lake originates from a lagoon that later turned into a freshwater lake. Examples of this sort are the lakes to the north and east of Ephesos (Stock et al., 2015) and Lake Azap in the hinterland of Miletos (Müllenhoff, Handl, Knipping, & Brückner, 2004). Three peat layers are interdigitated with the lake sediments. The development of peat requires a low water table, many plants (Meyers & Lallier‐Vergès, 1999; Meyers & Teranes, 2001), and anoxic conditions (Killops & Killops, 2005). Thus, the lake level must have considerably changed several times between the 4th and the 1st millennium BC, probably due to tectonics (see below). The lowest peat dates to the mid‐4th millennium BC, the medium peat originates from the end of the 3rd millennium to the beginning of the 2nd millennium BC, the upper peat dates to ca. 1000–500 BC. In the center of the Finike plain, Öner (2013) describes two peat layers that are separated in the western part where the lower one has a thickness of up to 5 m. A peat layer is a sign that the lake started to silt up; its covering by limnic strata documents another expansion of the lake.

In the cores between the eastern and the western city, a cultural layer was detected *grosso modo* at the same depth of 1.15–1.00 m b.s.l. It will never be unearthed since the water table starts already at a depth of 0.50 m b.s. due to the perennial discharge of the karstic springs. The finds of a sharp, angular silex, processed stones, and nonlocal big rounded stones are evidence of an intentional setting. This layer is on top of sediments which can be interpreted as representing the shore of a lake. The coring evidence is not sufficient to interpret this situation as the place of an early settlement in the second half of the 2nd millennium BC (dating to 1389–1115 BC and before 1230–1028 BC; Figure 5), but it certainly indicates a temporary use by humans.

Fluvial strata with sand and pebbles support the assumption that the lake started to silt up (the coring sites of Lim 12 and 16–19 are located at the western edge of the lake; Figure 11). At the latest between 1000 and 500 BC, the uppermost peat and once again lacustrine sediments were deposited. By then, the direct surroundings of the later city of Limyra must have been abandoned. However, the few drill cores can only give a first insight into the landscape formation.

Due to the fact that no drill cores have been retrieved from the eastern part of the Finike plain, the size of the lake could not be reconstructed. However, older scenarios of Öner (2013) can be taken into account. Many of his drill cores also reveal several meters of lake sediments. Uyanık et al. (2013), who studied the effects of liquefaction in the area of Kumluca in the eastern part of the Finike plain (see Figure 1 for location), confirm that lacustrine sediments are also present in that area. Therefore, the maximum lateral extension of the lake must have been at least 8–10 km.

The sediment cores close to the present coastline show fluvial sediments at the base, which are overlain by homogeneous sands that may originate from dunes (Figure 9). Öner (2013) proves the coastline with the beach barrier close to the location of Lim 20 until the mid‐2nd millennium BC (Figure 11), wherefore the shores of the lake must be located further to the north of this drill site.

#### 7.1.2 Landscape formation since the 1st millennium BC

Since the second half of the 1st millennium BC, several small river channels have been flowing through the Limyra area, gradually silting up the lake (Figure 11). The sediments originate from the mountains to the north. Today, the river Limyros is fed by several perennial springs from Toçak Dağı. Corings Lim 3, 8, 9, and 12 unearthed pebbles as a sign for river channel activities, whereas the other corings are dominated by sand which probably reflects the levees and inundation parts during floodings. Since the sand is void of organic material, ^14^C dating was not possible. It seems that the river channels often changed their courses.

When most of the former lake area was silted up, people started to settle. The settlement layers have a thickness of up to 3.50 m (Lim 4). The ^14^C age estimate in Lim 4 dates to the 6th–4th centuries BC which is in line with the foundation of the settlement during the 6th century BC and the first heyday in the 4th century BC (see Section 3). However, since the oldest archaeological finds date from the 7th century BC, at least the western area must have been suitable for settling already during that time, when some parts of the eastern city (Lim 10 and 3) were still swampy. There, the layers with human footprint have a thickness of only 0.50–1.50 m. With the rising groundwater level, presumably due to coseismic subsidence that may have been one reason for the abandonment of the settlement, peat started to grow and fluvial as well as alluvial sediments covered the former city area.

### 7.2 Sea‐level development, tectonics, and climate changes

For a better understanding of the formation of the Finike plain, the changes of the sea and groundwater levels, as well as climate changes, have to be taken into account. The Finike plain with the city of Limyra is located in a tectonically active region between the Hellenic and the Cyprus arcs and—as stated above—has been formed due to subsidence during the Pliocene‐Quaternary (Aksu et al., 2009; Hall et al., 2009). This could also be proven by Desruelles et al. (2009) who quantified the relative sea‐level evolution in this region. On the other hand, riverine and littoral deposition overcompensated the subsidence effect so that the shoreline prograded seawards.

The subsidence still continues and can directly be connected to the development of the relative sea and groundwater levels. The sudden rise in the lake level, which occurred two to three times during the last 5000 years as evidenced by the limnic sediments covering the peat layers, may go along with subsidence, which may have been triggered by strong earthquakes. The Finike earthquake of 1926 with 6.8 Mw clearly shows the tectonic activity of the region (Uyanık et al., 2013). For the coastline to the west of Finike, Anzidei et al. (2011) calculated an average subsidence of 1.48 mm/year and a sea‐level rise of up to 2.2 mm/year for the last 2300 years, whereas Kızıldağ et al. (2012) published a sea‐level rise of only up to 1 mm/year for the same time span. Desruelles et al. (2009) estimate a subsidence of up to 4.3 m since Roman times by evidence of a submerged beachrock near the mouth of Alakır Çayı. Lately, Mauz, Vacchi, Green, Hoffmann, and Cooper (2016) showed that beachrock can be used as sea‐level marker. At Andriake, to the west of Finike, Fouache, Sibella, and Dalongeville (2005) observed the submergence of a Roman quarry.

It is obvious that one factor for the evolution of the lake was the subsidence of the plain. Even before the Roman period, earthquakes must have occurred and influenced the local landscape formation. Therefore, the events which are documented in this study by the abrupt covering of the peat layers by lake sediments may be related to earthquake‐induced subsidence, which triggered a sudden expansion of the lake. Another example of a Holocene lake evolution in an active tectonic region is Lake Marmara in the Gediz Graben (Bulkan, Yalçın, & Wilkes, 2018)

For the environs of Finike, only few studies exist concerning the local sea‐level evolution. Desruelles et al. (2009) assume a relatively stable coastline in the Finike area in the last 2000 years. From the 2nd century BC to the 6th century AD, the authors presume the sea level at −1.5 m due to local subsidence. Studies from tectonically stable areas in the Mediterranean reveal that sea level was close to the present one already at the beginning of the 2nd millennium BC (Galili, Zviely, & Weinstein‐Evron, 2005) and ±0.50 m from the Middle Bronze Age to Medieval times (Porat, Sivan, & Zviely, 2008). However, for Elaia, the maritime satellite city of Pergamon, Seeliger et al. (2017) showed that sea level was ca. 1.75 m deeper than today at the turn of the eras. Brückner and Kelterbaum (2013) demonstrated that in the eastern Mediterranean the local tectonics often overprint the glacio‐eustatic changes.

Under the assumption that the peat layers of the former lake reflect the former lake level which in turn was closely connected to sea level, one can state the following regarding the paleogeography in the environs of Limyra: when these peats grew their position mirrored more or less the sea level. Terrestrial limits of these kinds of peats are dominated by freshwater fauna (Vacchi et al., 2016). The abrupt covering of the peats by lake sediments indicates sudden subsidence—most probably triggered by earthquakes. Considering the present position of the three peats and their ^14^C age estimates in coring Lim 12, the relative sea level was at −5.90 m around 3700–3500 BC, at −2.30 m around 2100–1800 BC and at −1.00 m around 1050–900 BC (see Figure 5). This is clear evidence of the strong influence of subsidence tectonics in the Limyra area. A systematic study and dating of the peat layers will decipher both the sea‐level and the earthquake histories in more detail.

Another factor that might be taken into account is the climate. During the Holocene, the three rapid climate change events (RCC) 8.2, 6.2, and 3 ka BP (Mayewski et al., 2004; Rohling, Mayewski, Abu‐Zied, Casford, & Hayes, 2002) resulted in cold and dry air masses in the Eastern Mediterranean. Weninger et al. (2014) as well as Kaniewski et al. (2017) studied the impact of the RCC on the civilizations of the Eastern Mediterranean. They may have been one reason for the end of the Aegean Bronze Age (Weninger et al., 2014). As for Limyra, the uppermost peat layer may correlate with the 3 ka BP event. However, in the Finike plain, we deal with a karstic water regime, which is not sensitive to climate fluctuations. The rivers, which are fed by karstic springs, flow all year long with a balanced discharge, even after the dry summers. Thus, the factor climate cannot have been of any importance.

## 8 CONCLUSION

For this research, 21 drill cores were carried out in the ancient city of Limyra and its environs. The study gives new insights into the landscape changes of the Finike plain since the Mid‐Holocene. The results reveal that a lake, which had probably evolved from a former lagoon, dominated the Finike plain for several millennia. More research is needed to decipher its origin and size. Embedded in the limnic sediments are up to three peat layers showing the repeated onset of the siltation of the lake. That they are all covered by lacustrine strata can be explained by rapid coseismic subsidence. Between the middle and the end of the 2nd millennium BC, a layer with anthropogenic remains (silex, processed stone, nonlocal big pebbles) at a depth of about 1.15–1.00 m b.s.l. was deposited in cores located between the western and the eastern city. Obviously, people made use of the lakefront. However, an event must have occurred leading to another rise in the lake level. During the 1st millennium BC, the lake completely silted up, and fluvial accumulation with shifting river channels began to dominate. People started to settle this area in the 7th century BC. As far as we know, the eastern city was abandoned between AD 1000 and 1200 due to the rising groundwater table, possibly caused by another earthquake. Then, peat grew and alluvium was accumulated. The formation of the Limyra area is a complex interplay between tectonics, lake evolution, river dynamics, sea‐level changes, and human‐environment interactions. Additional studies are needed to better understand and date the paleogeographic evolution of the Finike plain in general and the environs of Limyra in particular.
